# Uptake of an App-Based Case Management Service for HIV-Positive Men Who Have Sex With Men in China: Process Evaluation Study

**DOI:** 10.2196/40176

**Published:** 2023-04-26

**Authors:** Xiaoyan Fan, Ke Ning, Cong Liu, Haidan Zhong, Joseph T F Lau, Chun Hao, Yuantao Hao, Jinghua Li, Linghua Li, Jing Gu

**Affiliations:** 1 Department of Medical Statistics, School of Public Health, Sun Yat-sen University Guangzhou China; 2 School of Public Health, Li Ka Shing Faculty of Medicine, The University of Hong Kong Hong Kong SAR China; 3 Infectious Disease Centre, Guangzhou Eighth People's Hospital Guangzhou China; 4 Centre for Health Behaviors Research, School of Public Health and Primary Care, Faculty of Medicine, The Chinese University of Hong Kong Hong Kong Hong Kong; 5 Centre for Medical Anthropology and Behavioral Health, Sun Yat-sen University Guangzhou China; 6 Sun Yat-sen Global Health Institute, Sun Yat-sen University Guangzhou China; 7 Centre for Health Information Research, Sun Yat-sen University Guangzhou China; 8 Department of Epidemiology and Biostatistics, School of Public Health, Peking University Beijing China

**Keywords:** process evaluation, mobile health, mHealth, HIV, men who have sex with men, MSM, antiretroviral treatment, ART, case management

## Abstract

**Background:**

Men who have sex with men (MSM) in China are disproportionately affected by the HIV epidemic, and medication adherence to antiretroviral treatment in this vulnerable population is suboptimal. To address this issue, we developed an app-based case management service with multiple components, informed by the Information Motivation Behavioral skills model.

**Objective:**

We aimed to conduct a process evaluation for the implementation of an innovative app-based intervention guided by the Linnan and Steckler framework.

**Methods:**

Process evaluation was performed alongside a randomized controlled trial in the largest HIV clinic in Guangzhou, China. Eligible participants were HIV-positive MSM aged ≥18 years planning to initiate treatment on the day of recruitment. The app-based intervention had 4 components: web-based communication with case managers, educational articles, supportive service information (eg, information on mental health care and rehabilitation service), and hospital visit reminders. Process evaluation indicators of the intervention include dose delivered, dose received, fidelity, and satisfaction. The behavioral outcome was adherence to antiretroviral treatment at month 1, and Information Motivation Behavioral skills model scores were the intermediate outcome. Logistic and linear regression was used to investigate the association between intervention uptake and outcomes, controlling for potential confounders.

**Results:**

A total of 344 MSM were recruited from March 19, 2019, to January 13, 2020, and 172 were randomized to the intervention group. At month 1 follow-up, there was no significant difference in the proportion of adherent participants between the intervention and control groups (66/144, 45.8% vs 57/134, 42.5%; *P*=.28). In the intervention group, 120 participants engaged in web-based communication with case managers and 158 accessed at least 1 of the delivered articles. The primary concern captured in the web-based conversation was the side effects of the medication (114/374, 30.5%), which was also one of the most popular educational articles topics. The majority (124/144, 86.1%) of participants that completed the month 1 survey rated the intervention as “very helpful” or “helpful.” The number of educational articles accessed was associated with adequate adherence in the intervention group (odds ratio 1.08, 95% CI 1.02-1.15; *P*=.009). The intervention also improved the motivation score after adjusting for baseline values (β=2.34, 95% CI 0.77-3.91; *P*=.004). However, the number of web-based conversations, regardless of conversation features, was associated with lower motivation scores in the intervention group.

**Conclusions:**

The intervention was well-received. Delivering educational resources of interest may enhance medication adherence. The uptake of the web-based communication component could serve as an indicator of real-life difficulties and could be used by case managers to identify potential inadequate adherence.

**Trial Registration:**

Clinicaltrial.gov NCT03860116; https://clinicaltrials.gov/ct2/show/NCT03860116

**International Registered Report Identifier (IRRID):**

RR2-10.1186/s12889-020-8171-5

## Introduction

### Background

In China, men who have sex with men (MSM) are disproportionally affected by the HIV epidemic and remain one of the vulnerable populations susceptible to HIV infection via sexual transmission. The pooled prevalence of HIV among Chinese MSM from 2001 to 2018 was 5.7% (95% CI 5.4-6.1) [1], which was 50 times that of the general population in 2018 [2]. It was estimated that up to 53% (51%-56%) of MSM in China had unprotected sex with regular male partners [3]. Treatment as prevention has been identified as an effective strategy to control the HIV epidemic, and adherence to antiretroviral treatment (ART) is necessary to maintain an undetectable viral load among people living with HIV, thus reducing infection risk via sexual transmission [4]. However, despite the disproportional HIV epidemic and common transmission of HIV via sex among MSM in China, their ART adherence has been suboptimal with the proportion of adherent MSM on ART ranging from 69.1% to 83.6% [5-7]. The adherence rates fail to meet the *90-90-90* strategy announced by The Joint United Nations Programme on HIV and AIDS (UNAIDS) in 2014, which advocates that 90% of those on treatment achieve sustained viral suppression [8]. Therefore, interventions to improve and maintain medication adherence among HIV-positive MSM are needed.

The upsurge in mobile phone coverage and internet users in the past decade in China makes telemedicine and mobile health intervention highly feasible, which also applies to MSM, among whom the possession of cell phones and access to the internet is high [9]. In an effort to boost adherence to ART among MSM, our team developed a multicomponent case management service combining a health care service app, Trusted Doctor, and an instant messaging app, WeChat (Tencent Holdings Ltd), guided by the Information Motivation and Behavioral skills (IMB) model. It was hypothesized that the components of our intervention could improve or maintain medication adherence among MSM by increasing their HIV-related knowledge and enhancing their motivation, leading to better behavioral skills. By subscribing to the official account of Trusted Doctor via the WeChat platform, patients can communicate and interact directly with certified health care providers and can also receive educational articles, hospital visit reminders, and retrieve information on supportive services (eg, mental health and rehabilitation services) [10].

### Literature review

Multicomponent app-based interventions are increasing in popularity [11,12]. Previous studies have incorporated a variety of adherence-supporting features, such as personalized feedback, data visualization, reminders, educational information, and self-monitoring functions [12]. In 2015, the Medical Research Council (United Kingdom) published guidelines for process evaluation research targeting interventions with multiple components to better understand the mechanisms of complex interventions and facilitate the interpretation of trial results [13]. Most studies that attempted to conduct process evaluation for app-based behavior-changing interventions simply reported app use (eg, the number of clicks or views) and qualitative feedback of user experience (eg, barriers and satisfaction) without guidance from a theoretical framework or the exploration of the potential mechanism of action from a quantitative perspective [14-18]. In the field of app-based interventions for patients with HIV, we identified 3 process evaluation studies, of which 2 were pilot studies with small sample sizes (ie, 40 and 60 participants) [19,20], and 1 accompanied a cohort study with a larger sample size (N=462) [21]. The latter process evaluation was conducted for an adherence-supporting app among patients with HIV in the Philippines, guided by the Linnan and Steckler [22] framework. However, in their study, the effect of the intervention was not reported, as all participants received the intervention and the mechanism of action was not investigated.

### Aim

Therefore, in this study, we aimed to conduct a process evaluation of our multicomponent app-based intervention from a quantitative perspective. We first examined the effect of the intervention by comparing medication adherence and IMB scores between the intervention and control groups and then described the intervention uptake of each component guided by the Linnan and Steckler [22] framework, investigated the association between key components of the intervention with medication adherence and IMB scores, and eventually examined the potential mechanism of action among HIV-positive MSM.

## Methods

### Participants

Process evaluation was conducted alongside an ongoing open-label, parallel-group randomized controlled trial (RCT) in the HIV clinic in Guangzhou Eighth People’s Hospital in Guangzhou, China. Participants were recruited from March 19, 2019, to January 13, 2020. Eligible participants (1) were HIV-positive MSM aged ≥18 years, (2) had planned to initiate ART on the day of recruitment, and (3) had access to a personal smartphone and a private WeChat account to receive follow-up questionnaire links and the app-based intervention for the intervention group. More details regarding the recruitment procedure are described in a published protocol [10].

### Intervention

#### Overview

Eligible participants completed a baseline questionnaire on a tablet with assistance from field investigators and were then randomly assigned to the intervention or control group. The control group received a standard case management service, including a 20-minute education session before initiating ART and 4 hospital visits at months 1, 2, 3, and 6 for prescription refill, physical assessment, and meeting with case managers. Case managers would discuss the appointment schedule with each participant individually, and patients can reach case managers via phone calls during office hours to consult treatment-related concerns and questions or rearrange the appointment. The intervention group received the above standard service plus the app-based intervention, which consisted of the following 4 components, including web-based communication with case managers, educational articles delivery, supportive service information retrieval, and hospital visit reminders. The details of each intervention component are described in subsequent sections.

#### Rationale and Theoretical Framework of the Intervention

The design of the intervention was guided by the IMB model. It hypothesizes that individuals who are well-informed, motivated to act, and possess the necessary behavioral skills for effective action will be more likely to initiate and maintain healthy behaviors [23]. On the basis of this framework, we further hypothesized that a better connection to care (by sending hospital visit reminders and providing supportive service information) would also exert a positive influence and contribute to a better treatment outcome, such as adherence to ART (Figure S1 in Multimedia Appendix 1). After interviewing different stakeholders, including MSM on ART, engineers, case managers, and doctors, we proposed corresponding theoretical modules and ascertained the intervention components (Table S1 in Multimedia Appendix 1). A detailed intervention delivery schedule is documented in Multimedia Appendix 1 (Tables S2 and S3).

#### Web-Based Communication

Web-based communication was achieved via a combination of an instant messaging app (WeChat) used by the patients and the Trusted Doctor app used by the case managers. The patients can initiate conversations or receive messages via the WeChat platform connected to the Trusted Doctor app. To avoid overburdening case managers, we set a limit that up to 5 messages could be initiated in 3 days by patients as default. Case managers were authorized to adjust the limit after the primary evaluation of the patient’s situation. One-on-one training on the use of the app was provided to all the case managers.

#### Educational Articles Delivery

A total of 44 articles were sent in a prespecified order during the trial, of which 21 articles were sent during month 1. The delivered articles covered 12 themes and were categorized as primary and secondary according to the level of interest expressed by HIV-positive MSM on ART during the design phase. The primary category includes general introduction of the disease and treatment, tips about adherence, side effects of medication, and daily life adjustments. The secondary category includes psychological adaptation, disclosure to family or friends regarding one’s infection, and transmission prevention. Article titles, categories, and dates of delivery are detailed in Table S2 in Multimedia Appendix 1.

#### Supportive Service Information

We embedded a set of supportive services on the WeChat platform, on which the patients could access the information by clicking on relevant tabs. The following 5 tabs were provided: guidance on government reimbursement, retrieval of a digital version of physical testing results, rehabilitation services, mental health services, and treatment seeking for regular diseases.

#### Hospital Visits Reminders

Four hospital visit reminders were scheduled at months 1, 2, 3, and 6 in the intervention group.

### Outcomes

#### Overview

As suggested by the World Health Organization (WHO) guideline for ART, the first month of ART is especially important because immune reconstitution inflammatory syndrome and early adverse drug reactions can become potential barriers for patients to establish adequate adherence [24]. Therefore, after month-1 hospital visits, we conducted a process evaluation of the intervention uptake during month 1 and examined their associations with medication adherence (behavioral outcome) and IMB scores (intermediate outcome) at month 1.

#### Behavioral Outcome

Adherence to ART medication was measured using a validated 3-item scale with high sensitivity to inadequate adherence [25]. Participants who reported “no missing” pills, “always” taking pills as instructed, and rated their pill-taking performance as “excellent” in the last 30 days were categorized as having adequate medication adherence.

#### Intermediate Outcome

Information, motivation, and behavioral skills were measured using the *Life Windows Information–Motivation–Behavioral Skills Antiretroviral Therapy Adherence Questionnaire* (LW-IMB-AAQ), which was shown to have good validity and reliability in a Chinese sample of HIV-positive individuals undergoing ART [26,27]. The instrument consists of 33 items rated on a 5-point Likert scale, with 9 measuring information, 10 measuring motivation, and 14 measuring behavioral skills (the first item was excluded as it measured barriers according to the questionnaire guidance). Summative scores were derived for each construct, with a higher score reflecting better information, motivation, and behavioral skills.

We also collected information and motivation scores at baseline but did not collect behavioral skills, as it asked pill-taking scenarios (eg, “How hard or easy is it for you to take your HIV medications when your usual routine changes, for example, when you travel or when you go out with your friends?”), which does not apply to our participants who had not initiated the treatment at the time of the survey.

### Process Evaluation

#### Overview

Process evaluation was guided by the Linnan and Steckler [22] framework for complex public health interventions. Data on the following indicators for each intervention component were collected and reported: dose delivered, dose received, and fidelity. Overall satisfaction with the intervention was measured in the month-1 survey for the intervention group, whereas contamination was assessed in the month-1 survey for the control group. Dose delivered is defined as the amount of the intervention component delivered. Dose received refers to the extent to which participants actively interact with delivered resources or materials. Fidelity is defined as the quality or integrity of the intervention provided as planned. According to Linnan and Steckler [22], dose delivered and fidelity reflect the efforts of intervention providers, whereas dose received indicates the engagement of participants.

In this study, educational articles delivery, instructive message for supportive service information retrieval, and hospital visit reminders were automatically sent by the app, and their dose delivered was reported. Web-based communication and access to educational articles required interaction with participants, and the dose received was reported. Moreover, the web-based communication service was provided by case managers, and its fidelity was reported. Individual-level data on web-based communication and educational articles were provided by the Trusted Doctor company, whereas data on supportive service information can only be obtained via WeChat, in which only summative data are available. Therefore, web-based communication and educational articles were regarded as major intervention components and were primarily described and analyzed. A summary of the intervention component, corresponding process evaluation indicators, data sources, and type of data (ie, individual or summative) are listed in Multimedia Appendix 1 (Table S4).

#### Web-Based Communication

Dose received was assessed based on the average number of messages received and sent by the participants. Trusted Doctor provided deidentified granular data for web-based communication: the content, time stamp, and identification number of the participants or case managers who sent or received the message. Table S5 in Multimedia Appendix 1 details the criteria for eligible messages and the rules for coding them into dialogues. A dialogue refers to the set of messages related to an original inquiry followed by successive replies, which could be initiated by either case managers or participants [28].

As the assessment of fidelity can be flexible by definition, we developed a web-based patient-provider communication framework (Figure S2 in Multimedia Appendix 1), which integrates the core communication component (ie, communicative content) from the framework by Ong et al [29], timeliness, completeness, and style of the conversation. The framework by Ong et al [29] suggests that communicative behaviors (practical or emotional, ie, task-oriented or socioemotional) between doctors and patients could influence patients’ outcomes such as compliance. We further proposed other characteristics of web-based conversation, namely, the timeliness of the provider’s reply (whether the initial message of a conversation is replied to within a predefined period), completeness (whether patients’ questions are solved or referred to other professionals), and style (whether emoticons and formal language are used) [30-32]. Further details are provided in Table S5 (Multimedia Appendix 1). Dialogues were used as the minimal unit to report fidelity.

#### Educational Articles Delivery

For educational articles, the dose delivered and received were reported. During month 1, 21 selected articles were delivered to all participants in the intervention group. As data on whether the participants unsubscribed to the platform were not available, we presumed that all the articles were delivered as planned.

We used access to educational article links as a proxy for reading behavior. The dose received was measured by the number of access and access:delivery ratio (the number of access divided by the number of articles delivered) by article theme. Reading behaviors for primary and secondary categories were also constructed to assess the engagement of the participants. Reading behaviors were categorized as very high reading (ie, accessing every article delivered in month 1 at least once), high reading (ie, accessing only some of the articles with access:delivery ratio >1), adequate reading (ie, accessing only some of the articles with access:delivery ratio <1), and no reading engagement (not reading any of the articles in the specific category).

#### Supportive Service Information Retrieval

One reminder was automatically sent to all participants in the intervention group during the first month to inform them of the availability of the supportive service information embedded in the platform. Similar to the dose delivered by educational article delivery, dose delivered of supportive service information was presumed to be delivered as planned.

The dose received of supportive service information was measured by the number of clicks on the supportive service tab on the WeChat platform. As individual-level data were not available, only the overall number of clicks in each tab was reported.

#### Hospital Visit Reminders

The number of automatic reminder messages was reported as dose delivered, which was sent once in the first month.

#### Satisfaction

At the month-1 survey, the intervention group was asked to rate how helpful the intervention was to their treatment and life on a 5-point scale, the options varying from “not helpful at all” to “very helpful.”

#### Contamination

Contamination in the control group was measured by asking whether they engaged in web-based communication with research assistants (where the contact was established for delivering follow-up questionnaires) and whether they subscribed to other platforms for educational articles.

### Data Analysis

Descriptive statistics were generated for baseline variables, month-1 intermediate outcome (IMB scores), month-1 medication adherence, and indicators of process evaluation. Chi-square analysis and independent group 2-tailed *t* tests were used to compare adherence and IMB scores between groups, respectively. A pairwise 2-tailed *t* test was used to compare the change in information and motivation scores between baseline and month-1 follow-up.

In addition to descriptive analysis, we conducted a series of exploratory analyses to test the month-1 efficacy of the intervention and to explore the potential mechanism between intervention uptake and month-1 behavioral outcomes. The month-1 efficacy of the intervention was examined in the full sample using logistic and linear regression for binary (ie, adherence) and continuous outcomes (ie, IMB scores), respectively, adjusting for unbalanced baseline variables between groups. The applicability of the IMB framework to our study sample was tested using structural equation modeling for the full sample. We also divided the control group by contamination and repeated the above analysis as a sensitivity analysis. Subsequently, we investigated the potential intervention mechanisms by examining the association between intervention uptake (ie, dose received from web-based communication and educational articles delivery) and medication adherence or IMB scores. First, we ran logistic or linear regression between baseline characteristics and outcomes in the control group to identify potential confounders. Then, we examined how intervention uptake was associated with the outcome of interest, adjusting for identified confounders. Missing data were imputed using multiple imputations by chained equations. Both text analysis and data analysis were performed using the R software (version 4.1.0; R Foundation for Statistical Computing).

### Ethics Approval, Trial Registration, and Informed Consent

The trial was approved by the Ethics Committee of the School of Public Health, Sun Yat-sen University (approval #003 in 2017). The RCT was registered at ClinicalTrials.gov under registration number NCT03860116. Written informed consent was obtained from all participants before the baseline assessment. For data analysis, all personal identifiers were removed and replaced by a research ID in password-protected data files to ensure confidentiality.

## Results

### Overview

**Figure 1:**
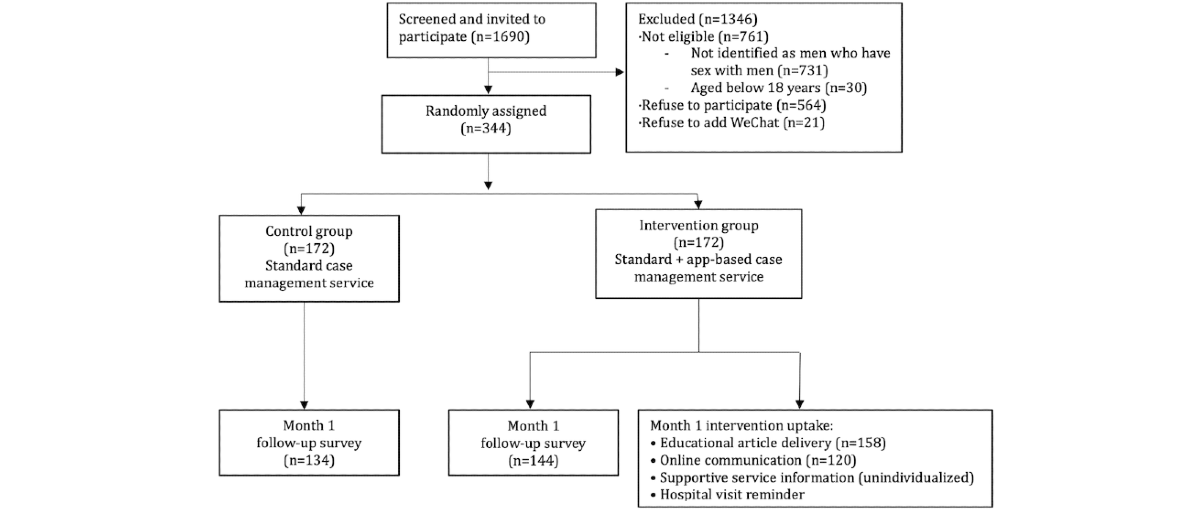
Flowchart of the process evaluation study.

Between March 19, 2019, and January 13, 2020, we screened a total of 1690 patients who attended the HIV clinic for the first time, of which 344 were eligible and agreed to participate in our study (Figure 1). All 172 participants were randomly assigned to the intervention group or the control group. At the month-1 follow-up, 144 and 134 participants from the intervention and control groups, respectively, completed the web-based survey.

### Baseline Characteristics

The baseline characteristics of the patients are presented in Table 1. Of all the participants, about two-thirds were young men (≤30 years; 205/338, 60.6%) and had a college degree or higher (218/344, 63.4%), and more than half (199/344, 57.8%) were single. Approximately half of the respondents worked in the private sector (157/344, 45.6%) and had a monthly income of ≥5000 Chinese Yuan (US $745; 192/344, 55.8%). The majority identified themselves as homosexual or bisexual (305/344, 88.7%), with the rest reporting their sexual orientation as heterosexual or unknown (39/344, 11.3%). Half (128/278, 46%) of the participants initiated their treatment with a CD4 cell count >350/µL. Most (287/344, 83.4%) of the participants used the free first-line ART regimen reimbursed by the government. The sociodemographic characteristics of our sample were consistent with prior serial cross-sectional surveys of MSM in Guangzhou [33].

At baseline, participants’ education level differed significantly between groups. Compared with the control group, the intervention group had a lower proportion of tertiary education (college degree or above, intervention vs control: 98/172, 57% vs 120/172, 69.7%; *P*=.04).

**Table 1:** Participants’ baseline characteristics (n=344).

Characteristics		All participants, n (%)	Control (n=172), n (%)	Intervention (n=172), n (%)	*P* value	
**Age (years; all participants: n=338; control: n=167; intervention: n=171)**						.75
	18-24	86 (25.4)	42 (25.1)	44 (25.7)		
	24-29	119 (35.2)	62 (37.1)	57 (33.3)		
	30	133 (39.3)	63 (37.7)	70 (40.9)		
**Education (all participants: n=344; control: n=172; intervention: n=172)**						*.04* ^a^
	Middle school or lower	55 (16)	25 (14.5)	30 (17.4)		
	High school	71 (20.6)	27 (15.7)	44 (25.6)		
	College degree	106 (30.8)	63 (36.6)	43 (25)		
	Bachelor’s degree or above	112 (32.6)	57 (33.1)	55 (32)		
**Employment (all participants: n=344; control: n=172; intervention: n=172)**						.52
	Students	43 (12.5)	24 (14)	19 (11)		
	Public sector	11 (3.2)	6 (3.5)	5 (2.9)		
	Private sector	157 (45.6)	82 (47.7)	75 (43.6)		
	Other	133 (38.7)	60 (34.9)	73 (42.4)		
**Monthly income (CNY, per 1 CNY=US $0.149; all participants: n=344; control: n=172; intervention: n=172)**						.08
	<1000	61 (17.7)	29 (16.9)	32 (18.6)		
	1000-5000	91 (26.5)	37 (21.5)	54 (31.4)		
	5000-10,000	103 (29.9)	61 (35.5)	42 (24.4)		
	>10,000	89 (25.9)	45 (26.2)	44 (25.6)		
**Marital status (all participants: n=344; control: n=172; intervention: n=172)**						.99
	Single	199 (57.8)	100 (58.1)	99 (57.6)		
	Not single	145 (42.2)	72 (41.9)	73 (42.4)		
**Sexual orientation (all participants: n=344; control: n=172; intervention: n=172)**						.73
	Homosexual or bisexual	305 (88.7)	151 (87.8)	154 (89.5)		
	Do not know or others	39 (11.3)	21 (12.2)	18 (10.5)		
**Baseline CD4 count (all participants: n=278; control: n=134; intervention: n=144)**						.10
	≥350	128 (46.0)	69 (51.5)	59 (41)		
	<350	150 (54.0)	65 (48.5)	85 (59)		
**Regimen (all participants: n=344; control: n=172; intervention: n=172)**						.99
	Free first-line regimen	287 (83.4)	144 (83.7)	143 (83.1)		
	Other regimens	57 (16.6)	28 (16.3)	29 (16.9)		

^a^Indicates significant between-group difference.

### Medication Adherence and Intermediate Outcomes

At the month-1 follow-up, 45.8% (66/144) of the participants in the intervention group and 42.5% (57/134) in the control group reported adequate adherence without statistical significance (*P*=.28). The intervention efficacy remained nonsignificant after adjusting for unbalanced sociodemographic variables (ie, education level; Table 2). The IMB scores at baseline and month-1 follow-up are presented in Table S6 and Figure S3 (Multimedia Appendix 1). There were no significant differences in the IMB scores between the intervention and control groups at both time points. However, the information scores decreased significantly from baseline to month 1 follow-up in both groups (*P*<.001). For motivation scores, the intervention group showed no difference between baseline and month-1 follow-up (*P*=.21), whereas the control group showed a significant decrease (*P*=.003).

**Table 2:** Effects of intervention on intermediate and health outcomes of the 2 groups.

Outcomes		OR^a^ or β coefficient (95% CI)		*P* value
**Health-related outcomes^b^**				
	Adherence	1.31 (0.80 to 2.13)	.28	
**Intermediate outcomes^c^**				
	Information score	−0.19 (−1.30 to 0.92)	.73	
	Motivation score	*2.34 (0.77* *to* *3.91)* ^d^	*.004*	
	Behavioral skills	0.93 (−1.23 to 3.10)	.40	

^a^OR: odds ratio.

^b^Indicates OR.

^c^Indicates β coefficient.

^d^Indicates significant effect size.

### Process Evaluation Indicators

#### Web-Based Communication

##### Dose Delivered

In total, 120 (70%) of 172 participants were engaged in web-based conversations with case managers, generating 1664 messages. On average, each participant who engaged in web-based conversation sent 7 messages and received 6 during month 1. After coding these messages into dialogues, one-third (37/120, 30.8%) had only 1 dialogue with case managers, and two-fifths (46/120, 38.3%) had 2 or 3 dialogues, with the rest having ≥3 dialogues.

##### Fidelity

**Figure 2:**
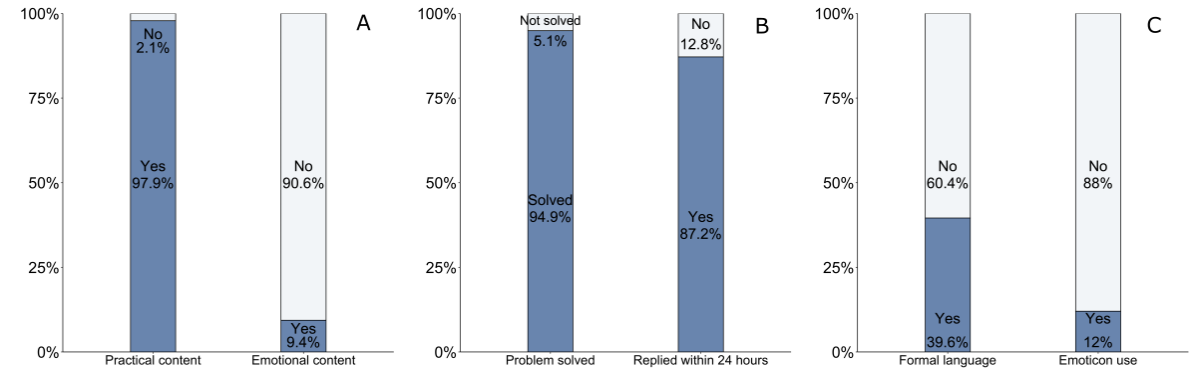
Characteristics of web-based patient-provider communication. (A) The content of the web-based conversation. (B) The completeness and timeliness of the web-based conversation reply. (C) The style of the web-based conversation (unit: dialogue).

A total of 401 dialogues were coded to assess the fidelity of web-based communication, including content (ie, practical or emotional), completeness (whether the questions were solved), timeliness (defined as whether the initial message was replied within 24 hours), and style (ie, formal language use and emoticon use; Figure 2).

Of the 401 dialogues, 374 (93.2%) received a text-based response and the rest responded with web links. Of the 374 dialogues, 366 (97.9%) had practical content focusing on problem solving, and the primary concern in web-based conversations was the side effects of medication (114/374, 30.5%). Other topics included changes in daily life habits because of treatment (87/374, 23.3%), hospital visit arrangement (66/374, 19.4%), the influence of imperfect medication-taking behavior (36/374, 9.4%), necessary physical examinations (32/374, 8.6%), other potential treatment regimens (13/374, 3.5%), transmission prevention (10/374, 2.8%), comorbidity (4/374, 1.3%), and other undefined topics (12/374, 3.2%). A small portion (35/374, 9.4%) of the dialogues involved emotional content, that is, patients expressed negative feelings or case managers tried to cheer up patients. Regarding completeness, 94.9% (355/374) of the issues were solved directly, whereas the rest were not solved or referred to other professionals. In terms of timeliness, the majority (326/374, 87.2%) replied within 24 hours. Regarding the style of web-based conversation, formal language was used in 39.6% (148/374) of the dialogues (eg, greetings before questions, patients’ expressions of gratitude, or case managers’ expression of willingness to help). Emoticon was less common, as it was used in only 12% (45/374) of the dialogues.

#### Educational Articles Delivery

**Figure 3:**
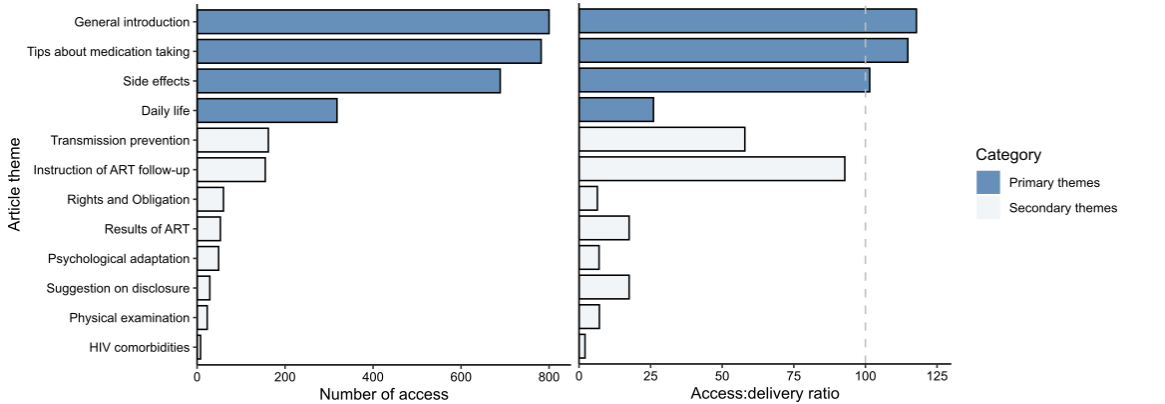
Dose received of the educational articles by theme. (A) The number of access for education articles of each theme. (B) Access:delivery ratio for articles of each theme. ART: antiretroviral treatment.

Of the 172 participants in the intervention group, 158 (91.9%) accessed at least 1 delivered article. The total number of accesses was 3128. Articles from the primary category were more popular than those from the secondary category (Figure 3), with an overall access:delivery ratio for the primary category (79.4%) 5 times that of the secondary category (16.6%). Themes that had the highest access and access:delivery ratio covered general introduction of the disease and treatment, tips about medication, and side effects of medication from the primary category, whereas the themes in the secondary category, including instruction of ART follow-up and transmission prevention, were also relatively popular with an access:delivery ratio of 92.8% and 57.9%, respectively. For articles from the primary category, 27.8% (44/158) of participants showed very high reading, 39.2% (62/158) showed high reading, and 32.9% (52/158) showed adequate reading engagement. In contrast, only 5.1% (8/158) participants showed high reading for articles from the secondary category, 81% (128/158) displayed adequate reading, and 13.9% (22/158) did not.

#### Supportive Service Information

The services tab named *Guidance on getting digital testing results* was the most popular service, with a total of 202 clicks. The other service tabs received a moderate number of clicks, ranging from 33 for *Rehabilitation service* to 98 for *Treatment seeking for regular diseases.*

#### Hospital Visit Reminders

A reminder was sent to each participant in the intervention group. In total, 172 reminder messages were sent.

#### Satisfaction

Out of 144 participants in the intervention group who completed the web-based survey, 47 (32.6%) rated the intervention platform to be “very helpful” and 77 (53.5%) rated as “helpful.”

#### Mechanism of Action of the Intervention

**Figure 4:**
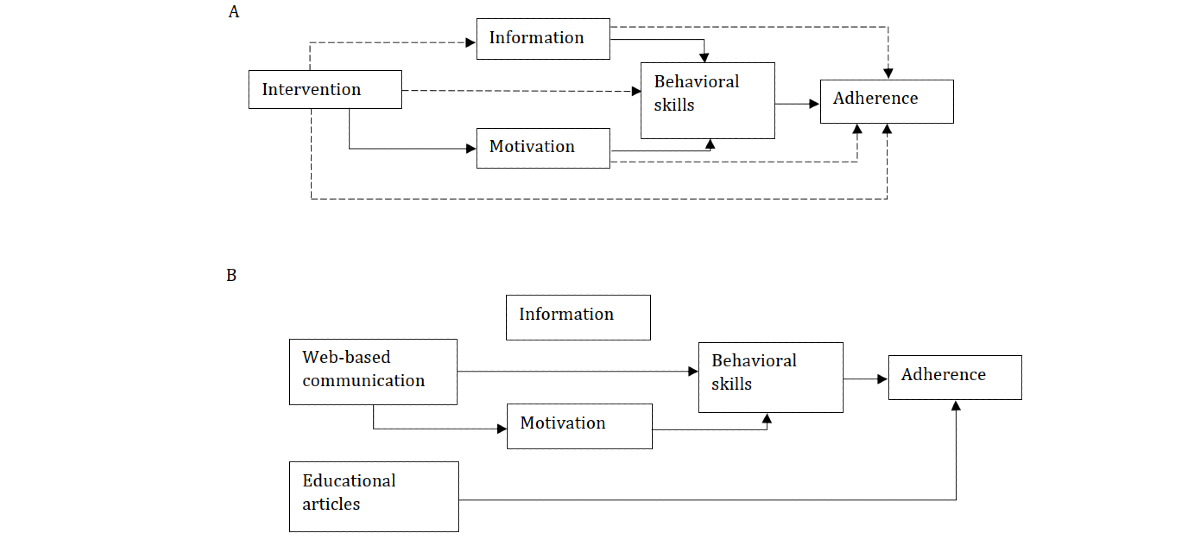
Hypothesis and tested associations about intervention mechanism. (A) Directed acyclic graph of the intervention mechanism in full sample. (B) Directed acyclic graph of the intervention components and outcomes in the intervention group. Each directed line represents a hypothesized causal relationship. The solid black line represents significant association tested. The dashed line represents nonsignificance.

Using structural equation modeling, we solidified the application of the IMB models to the full sample (Figure S4 in Multimedia Appendix 1). Thus, we examined the pathways between the intervention and control groups, as illustrated in Figure 4A. We did not detect significant differences in information scores and behavioral skills between the intervention and control groups, and the motivation scores of the intervention group were significantly higher (β=2.34, 95% CI 0.77-3.91) than those of the control group at the month-1 follow-up after adjusting for baseline scores (Table 2). The results did not change after considering contamination in the control group (Table S7 in Multimedia Appendix 1).

We also examined the relationship between intervention uptake (ie, web-based communication and educational articles) and IMB scores in the intervention group, as illustrated in Figure 4B. We found that the number of articles accessed, either all articles or articles in the primary category, was significantly associated with better adherence after adjusting for confounding factors (Table 3), but not with IMB scores (Table 4). In contrast, the use of web-based communication was not associated with adherence (Table 3), but a higher number of web-based communications, regardless of conversation characteristics, was associated with lower motivation scores, and more emotional content involved in web-based communication was associated with lower behavioral skills (Table 4). Education and monthly income were found to be associated with adherence in the control group and were therefore adjusted when investigating the association between intervention uptake and adherence in the intervention group (Table S8 in Multimedia Appendix 1). No confounding factors were adjusted for IMB scores, as none were detected in the control group (Table S9 in Multimedia Appendix 1).

**Table 3:** Association between intervention uptake and month-1 adherence in the intervention group. Logistic regression models adjusting for factors associated with adherence in the control group (ie, education and monthly income) were constructed to obtain odds ratio (OR) and 95% CI.

Variables						OR (95% CI)			*P* value
**Numbers of conversations**									
	**Content**								
		Practical support			0.98 (0.86-1.12)			.81	
		Emotional support			0.79 (0.54-1.16)			.23	
	**Completeness**								
		Problem solved			0.97 (0.84-1.12)			.70	
	**Timeliness**								
		Replied within 24 hours			0.97 (0.83-1.13)			.72	
	**Style**								
		Either side using emoticons			0.83 (0.55-1.25)			.37	
		Either side using formal language			1.01 (0.83-1.23)			.90	
**Number of articles accessed**									
	**Overall**				*1.08 (1.02-1.15)* ^a^			*.009*	
		**Primary category**			*1.11 (1.02-1.21)*			*.02*	
			General introduction	*1.45 (1.08-1.94)*			*.01*		
			Medication tips	*1.31 (1.01-1.7)*			*.048*		
			Side effects	*1.34 (1.04-1.71)*			*.02*		
			Daily life	1.22 (0.92-1.61)			.17		
		**Reading engagement of primary articles**							
			Inadequate reading	Reference			—^b^		
			Adequate reading	0.97 (0.41-2.28)			.94		
			Very high reading	0.43 (0.18-1.06)			.07		

^a^Odds ratios and *P* values that indicated significant effect size were italicized.

^b^Not applicable as inadequate reading was the reference level.

**Table 4:** Association between intervention uptake and scores of month-1 Information Motivation Behavioral skills model constructs in the intervention group. Baseline scores were adjusted in the regression model. For information scores, whether engaged in web-based communication was adjusted.

Variables					Information, β (95% CI)		Motivation, β (95% CI)		Behavioral skills, β (95% CI)
**Numbers of conversation**									
	**Content**								
		Practical support		–0.1 (–0.33 to 0.13)		–*0.7 (–0.99 to–0.4)*^a^		–0.32 (–0.75 to 0.11)	
		Emotional support		–0.37 (–0.79 to 0.05)		–*0.99 (–1.65 to –0.33)*		–*0.94 (–1.72 to –0.16)*	
	**Completeness**								
		Problem solved		–0.13 (–0.34 to 0.09)		–*0.72 (–1.03 to –0.42)*		–0.30 (–0.76 to 0.16)	
	**Timeliness**								
		Replied within 24 hours		–0.14 (–0.39 to 0.1)		–*0.73 (–1.04 to –0.42)*		–0.35 (–0.79 to 0.1)	
	**Style**								
		Either side using emoticons		0.02 (–0.47 to 0.51)		–*1.48 (–2.51 to –0.46)*		–0.67 (–2.2 to 0.86)	
		Either side using formal language		–0.03 (–0.34 to 0.27)		–*0.89 (–1.33 to –0.44)*		–0.15 (–0.77 to 0.47)	
**Number of articles accessed**									
	**Overall**				0.02 (–0.11 to 0.14)		–0.07 (–0.23 to 0.09)		0.05 (–0.21 to 0.31)
		**Primary category**		0.03 (–0.14 to 0.19)		–0.11 (–0.32 to 0.09)		0.09 (–0.26 to 0.43)	
			General introduction	–0.15 (–0.76 to 0.47)		–0.35 (–1.15 to 0.45)		0.27 (–0.97 to 1.52)	
			Medication tips	–0.05 (–0.61 to 0.52)		–0.2 (–0.9 to 0.49)		0.16 (–0.98 to 1.31)	
			Side effects	0.24 (–0.3 to 0.78)		–0.37 (–1.04 to 0.31)		0.62 (–0.45 to 1.68)	
			Daily life	0.05 (–0.44 to 0.55)		–0.29 (–0.92 to 0.34)		–0.33 (–1.35 to 0.69)	
		**Reading engagement of primary articles**							
			Inadequate reading	Reference		Reference		Reference	
			Adequate reading	–0.75 (–2.61 to 1.1)		–0.34 (–2.69 to 2.01)		0.80 (–2.71 to 4.31)	
			Very high reading	–0.35 (–2.25 to 1.55)		1.62 (–0.94 to 4.18)		0.48 (–3.35 to 4.31)	

^a^β coefficients and 95% CI that indicated significance was italicized.

#### Contamination

During the study period, 29.7% (51/172) of the participants in the control group initiated web-based communication with our research team via WeChat contact, and 51% (69/134) of participants in the control group reported subscribing to other WeChat platforms for educational materials.

## Discussion

### Principal Findings

In this study, we conducted a process evaluation of an app-based intervention for HIV-positive MSM. We found no statistically significant difference in medication adherence between the intervention and control groups at the month-1 follow-up. The intervention components were well received, especially web-based communication and educational articles delivery. The uptake of educational articles was associated with better adherence. Guided by the IMB model, we found that the app-based intervention helped maintain the motivation score among the participants, whereas the motivation score showed a decreasing trend in the control group. However, web-based communication was associated with lower motivation scores in the intervention group.

Detecting null results of medication adherence between the intervention and control groups is not uncommon in app-based interventional studies, in which studies investigate the efficacy of an app in addition to usual care, as in this study. In a small (N=50) RCT conducted among HIV-positive people with substance use disorder, DeFulio et al [34] designed an app that required participants to submit video selfies of medication consumption with monetary incentives and provided useful resources (including educational articles about ART and adherence and a list of supportive community resources). DeFulio et al [34] found that adherence to ART was similar between the intervention and control groups at month 1 (intervention vs control, 48% vs 39%, *P*=.72), whereas at month 6, a significant effect of the intervention app was detected (*P*=.03) mainly from the decreasing adherence in the control group, but this effect was not maintained after adjusting for covariates (*P*=.09). In another study that aimed to boost pre-exposure prophylaxis adherence among young MSM and transgender women (n=200), youth-friendly services were provided to all participants [35]. The youth-friendly services provided ongoing counseling or support outside scheduled visits via web-based instant messaging or telephone calls, with responses provided within 24 hours. In addition, the intervention group received an app that included self-assessment of HIV acquisition risk, point rewards, and reminders for preexposure prophylaxis and clinic appointments. However, researchers found no between-group differences for both at month 3 (intervention vs control: 54% vs 51%, *P*=.64) and at month 6 (49% vs 44%, *P*=.54). Mixed results were also commonly observed in studies (sample sizes ranged from 28 to 227) that compared a standard app with an augmented app intervention, in which a richer spectrum of features was embedded [36-38].

Compared with previous studies, we hypothesized that one of the reasons for our null result may be the so-called *honeymoon period* of good adherence, especially when our participants were HIV-positive MSM who just initiated ART [39]. Thus, it may take longer than 1 month for the adherence-supporting effect of our intervention to materialize. For example, in the control group, approximately 90% of the participants reported no missing pills, over 90% rated the medicine-taking behavior as *good* or *very good* or *excellent*, and over 95% reported *usually* or *most of the time* or *always* taking pills as instructed in the past month. This good adherence may decline over time, as mentioned in the study by DeFulio et al 34]. This hypothesis could also be supported by our findings that the motivation to adhere to ART saw a significant decreasing trend in the control group from baseline to month 1, in contrast with an increasing trend (though not statistically significant) in the intervention group (Figure S3 in Multimedia Appendix 1).

Key intervention components were well-received, with 69.8% (120/172) participants engaging in web-based communication and 91.9% (158/172) participants accessing educational articles. Most of the web-based conversations revolved around problem-oriented content, which echoes the most frequently visited themes of educational articles, such as medicine taking, side effects of ART medication, and hospital visits. Together with the decreases in information score in both groups (Figure S3 in Multimedia Appendix 1), our study emphasizes patients’ great need for reliable information resources and materials in the early phase of ART treatment to consolidate the 20-minute education session in standard case management services. The positive association between high reading engagement and better medication adherence also reflects the usefulness of reading educational articles to improve medication adherence.

Under the IMB framework, we examined the potential mechanisms of our key intervention components, and several unexpected findings are worth our attention. First, the hypothesis that the association between reading engagement and adherence is through the information-behavioral skills-adherence pathway (Figure S4 in Multimedia Appendix 1) was not validated, as the uptake of educational articles was not associated with either information score or behavioral skills in our study (Table 4). It is likely that reading engagement improved adherence in our study through other pathways, which is not measured by the IMB questionnaire. Second, it was found that motivation to adhere was decreasing in the control group, whereas it was stable, if not increasing, in the intervention group (Figure S3 in Multimedia Appendix 1); in contrast, higher use of web-based communication, regardless of the communication features, was associated with lower motivation scores in the intervention group. The majority (366/374, 97.9%) of the web-based conversations were practical content, which indicates that those patients were experiencing HIV-related problems, such as having side effects (eg, dermatitis), diet restrictions, and missing a pill. These difficulties could foster negative perception of the treatment, which was precisely captured by the motivation questionnaire (eg, *It upsets me that HIV medications can cause side effects or affect my look or hurt my health*)*.* In comparison, it was more likely that participants who did not engage with case managers were going through the initial adaptation period without outstanding side effects or other negative personal experiences. Therefore, the seemingly contradictory findings may indicate that the availability of web-based support to patients, not necessarily used, could provide a psychological buffer that maintains patients’ motivation to adhere; however, when a web-based conversation was initiated, it could signal that the patients were facing barriers that may lower their motivation and result in inadequate adherence in the long term. For those who frequently engage in web-based conversations, case managers should be alerted so that immediate support could be provided to solve their objective difficulties and avoid negative perceptions of the treatment.

It is encouraging that the majority (124/144, 86.1%) of participants in the intervention group rated the intervention as helpful or very helpful to their life in the month-1 survey. Nevertheless, the well-received web-based conversation cannot be achieved without the dedication of case managers. The timeliness of web-based conversation is an advantage of our app-based intervention compared with other prevalent contact channels, such as email. During our training for case managers, the timeliness of web-based conversation was emphasized, as previous studies showed that timely responses from health care workers for web-based inquiries were highly valued by patients [40,41]. Although the timeliness for web-based communication was achievable in our trial, incorporating such practice into the daily routine of case managers requires further consideration. HIV case management in China is still in its infancy, with the number of required case managers far exceeding that of the current staff. It was reported that <10 working case managers were responsible for >6000 patients with HIV in an HIV clinic in one of the most heavily HIV-affected provinces in China [42], failing to fulfill the requirement of 1 case manager for every 150 patients [43]. Under such an already demanding workload, reimbursement and ways to alleviate the stress because of potential increases in workload are needed to conquer providers’ reluctance to provide web-based consultations [44]. During informal communication with case managers, we learned that some case managers received small cash rewards sent voluntarily by patients as an expression of gratitude, which motivated the case managers and ameliorated their pressure from exposure to work. Hence, exploring a mutually beneficial web-based service model could be promising, such as cash compensation from the institution or voluntary service purchases from patients [45].

This study had several limitations. First, process evaluation can only be conducted at month-1 follow-up, which is short and may lead to our null findings. This was because of the discontinued collaboration with the technology company. Therefore, month-1 adherence, which can be used for investigating mechanisms, was used as a proxy for short-term efficacy measures. However, according to ART guidelines, the first month after ART initiation is critical for adequate adherence to medicine-taking behavior [24]. Conducting a process evaluation during this key phase is therefore informative in understanding the effective components of a complex intervention. Second, as we did not have individualized data for the uptake of supportive information retrieval or hospital visit reminders, associations between the uptake of these 2 components and adherence or intermediate outcomes could not be investigated. Third, we used the number of clicks on article links as a proxy for reading engagement in educational articles, which is likely to overestimate participants’ engagement. In addition, we could not assess the dose received by hospital visit reminders, as this component is a 1-way message, and the full attendance of month-1 visits in the intervention group could not reflect the dose received, as all participants in the control group had full attendance at the month-1 visit as well. Fourth, our findings might be subjective to false discovery, as a series of hypotheses were tested using exploratory analysis. Future studies are warranted to solidify our findings. Fifth, both groups could communicate with case managers via phone; however, this behavior was not assessed in our survey. Communication behaviors between the control group and our research assistants might be used as a proxy for attempted communication with case managers, but they were not examined and analyzed as thoroughly as in the intervention group. However, we conducted sensitivity analyses to compare participants who were contaminated with those who were not (Table S7 in Multimedia Appendix 1). Sixth, we did not conduct a formal qualitative analysis, as qualitative information was obtained only during casual discussions during fieldwork. Seventh, proficiency in using smartphones and the WeChat app was required to participate in this study, which might lead to selection bias and limit the generalizability of the findings. Previous studies suggest that susceptible populations, including older people and those with lower educational attainment, have similar service needs for web-based consultation but have less access to such services [46]. Accessible interventions should also be designed and implemented to target these populations. Finally, the web-based communication framework used in this study has not been validated, and psychometric validation studies are warranted in the future.

The strength of this study was the objective measures for intervention uptake rather than self-reported measures, which could yield more reliable evidence regarding the uptake of web-based conversations and reading of educational articles. In particular, web-based communication data in this study were collected and analyzed in a quantitative manner, guided by a self-designed framework. Previous studies on web-based communication usually included self-reported web-based communication behaviors or beliefs, and the findings were therefore subject to recall or measurement bias. The increasing popularity of web-based medical consultation in China [47], especially during the COVID-19 pandemic, calls for a theoretical basis for web-based patient-provider communication to accelerate our understanding of the potential benefits and mechanisms of web-based medical consulting services. Moreover, this study was based on the collaboration between researchers and health care providers in the clinic, where web-based communication services were provided by case managers. This means that our intervention was designed to be integrated as part of the HIV clinic’s routine service and could be transformed and embedded into routine health care for patients with HIV. In contrast, many intervention studies were conducted by researchers, which are not sustainable after the trial ends. Furthermore, previous studies that distributed educational articles did not account for the time-varying needs for education topics over the treatment period, whereas our delivery of educational articles was inspired by qualitative interviews with MSM who were already on ART and was delivered according to a predesigned order meeting their potential needs at specific periods.

### Conclusions

The intervention was well-received. Delivering educational resources may enhance medication adherence. The uptake of the web-based communication component could serve as an indicator of real-life difficulties and could be used by case managers to identify potential inadequate adherence.

## Appendix

Multimedia Appendix 1 The theoretical base and conceptual model of intervention design, details of intervention delivery for each component, supplementary methodology regarding data processing, and supplementary results.

## Abbreviations

ART: antiretroviral treatment
IMB: information, motivation, and behavioral skills
LW-IMB-AAQ: Life Windows Information–Motivation–Behavioral Skills Antiretroviral Therapy Adherence Questionnaire
MSM: men who have sex with men
OR: odds ratio
RCT: randomized controlled trial
UNAIDS: The Joint United Nations Programme on HIV and AIDS
WHO: World Health Organization

## Data Availability

The data sets used and analyzed during this study are available from the corresponding author upon reasonable request.
