# Supplementary material for: Uptake of an App-Based Case Management Service for HIV-Positive Men Who Have Sex With Men in China: Process Evaluation Study
Source: J Med Internet Res. 2023 Apr 26;25:e40176. doi: 10.2196/40176 (PMC10173030; doi:10.2196/40176)
Supplement: Multimedia Appendix 1 [file jmir_v25i1e40176_app1.docx]

**Supplementary Appendix**

Figure S1. Theoretical framework of intervention design. 1

Table S1. Innovative case management service model 2

Table S2. Delivery arrangement of educational articles 3

Table S3. Delivery arrangement of instruction messages on service retrieval within the platform 4

Table S4. Definition and reporting for process evaluation indicators 5

Figure S2. Online patient-provider communication framework 6

Table S5. Coding rules for online communication data 7

Table S6. Baseline and month-one Information Motivation and Behavioral skills model constructs in intervention and control group. 8

Figure S3. Changes of information and motivation scores from baseline to month one in two groups. 9

Figure S4. Results of structural equation modeling to test the IMB framework in the full sample 10

Table S7. Association between intervention and contamination and outcomes. 11

Table S8. Association between baseline variable and month one adherence in the control group. 12

Table S9. Association between baseline variable and month one month-one Information-Motivation-Behavioral skills (IMB) model constructs in the control group. 13

# Figure S1. Theoretical framework of intervention design.

**Adherence information**

🞄 Information about HIV/AIDS

🞄 Information about adherence

🞄 Information about side effects and drug interactions

**Adherence Motivation**

🞄 Attitude: beliefs and perceptions about non-adherence and adherence and relevant outcomes

🞄 Social norms: perceptions of support from others for adherence

**Adherence behavioral skills**

🞄 Objective skills to adhere

🞄 Perceived self-efficacy

**Adherence behavior**

**Psychosocial factors**

🞄 Mental health

🞄 Care connection

# Table S1. Innovative case management service model

| **Case management process** | **IMB constructs** | **Service content** | **Intervention components** |
| --- | --- | --- | --- |
| Assessment | Motivation | Establishing rapport | Communication |
| Plan | Motivation | Discussing treatment plan | Face-to-face communication |
| Service | Information | Knowledge regarding HIV/ART | Educational articles |
|  | Information | Knowledge regarding treatment process? | Educational articles |
|  | Information | Information about preventing transmission | Educational articles |
|  | Behavioral skills | Behavioral skills of adherence | Communication |
|  | Behavioral skills | How to promote partner testing | Communication |
|  | Behavioral skills | Avoidance of risky behavior | Communication |
|  | Mental health | Counseling | Communication |
|  | Care connection | STI treatment | Supportive service information |
| Coordination | Mental health | Psychological support | Supportive service information |
|  | Care connection | Abstinence therapy | Supportive service information |
|  | Care connection | Other social service | Supportive service information |
| Evaluation | Care connection | Tests results retrieval | Supportive service information |
|  | Care connection | Follow-up service | Hospital visit reminders |

# Table S2. Delivery arrangement of educational articles

| **Title** | **Category** | **Theme** | **Days of delivery** |
| --- | --- | --- | --- |
| *Answers to the ten big questions worrying you* | General introduction | Primary | 1 |
| *Things you need to know when you start ART* | General introduction | Primary | 2 |
| *Things you should keep in mind about different regimen* | Tips about medication taking | Primary | 3 |
| *Common questions that might arise during ART* | Tips about medication taking | Primary | 4 |
| *Let’s talk more about ART adherence* | Tips about medication taking | Primary | 5 |
| *How to deal with nausea and vomiting related to ART* | Side effects | Primary | 6 |
| *Care giver limitation and labor contract if you’re infected* | Rights and Obligation of PLWHA | Secondary | 7 |
| *How to live and cope with side-effects of medication* | Side effects | Primary | 8 |
| *Solutions about ART side-effects* | Side effects | Primary | 9 |
| *Necessary tips if you want to take meds properly* | Tips about medication taking | Primary | 10 |
| *Do you really KNOW this disease after 30 years* | General introduction | Primary | 11 |
| *How to solve sleeping disorders after initiating ART* | Side effects | Primary | 12 |
| *What can you eat if you’re on ART* | Daily life | Primary | 13 |
| *Mental suggestion for people who are newly infected* | Psychological adaptation | Secondary | 14 |
| *Sixteen major questions about A* | General introduction | Primary | 17 |
| *How to prevent transmission: is there anything I can do more that safe sex?* | Transmission prevention | Secondary | 19 |
| *Why can’t I ask someone else to pick up my pills?* | Instruction of ART follow-up | Secondary | 21 |
| *Ask an expert: should tell someone else about my status* | Suggestion on disclosure | Secondary | 24 |
| *Mental health issues to notice about A* | Psychological adaptation | Secondary | 26 |
| *How to arrange physical exams after ART initiation* | Physical examination knowledge | Secondary | 28 |
| *How to read a physical examination report?* | Physical examination knowledge | Secondary | 31 |
| *Daily life tips if you’re on ART* | Daily life | Primary | 33 |
| *Daily meds you shouldn’t take together with ART regimen* | Daily life | Primary | 38 |
| *Border entry policies for A-bies from 131 countries and areas.* | Rights and Obligation of PLWHA | Secondary | 40 |
| *Rights protection for A-bies* | Rights and Obligation of PLWHA | Secondary | 47 |
| *How to get along with clinician* | Rights and Obligation of PLWHA | Secondary | 54 |
| *How do I know if the regimen works* | Results of ART | Secondary | 61 |
| *Laws and regulations about sexual relationship and marriage.* | Rights and Obligation of PLWHA | Secondary | 68 |
| *One picture to help you tell the depressed status from depression.* | Psychological adaptation | Secondary | 75 |
| *When should I change my regimen* | Results of ART | Secondary | 78 |
| *How to deal with a fever if I am on ART* | Daily life | Primary | 82 |
| *Watch out: opportunistic infection* | HIV co-morbidities | Secondary | 96 |
| *Things you have to know about commercial insurance if you are on ART* | Rights and Obligation of PLWHA | Secondary | 103 |
| *Which vaccines you can use while on ART* | Daily life | Primary | 110 |
| *Tips for travelling while on ART* | Daily life | Primary | 117 |
| *Mental disorders make half of your disease, says Zhong Nanshan* | Psychological adaptation | Secondary | 124 |
| *How is A related to sexually transmitted diseases?* | HIV co-morbidities | Secondary | 131 |
| *Solutions to common opportunistic infections* | HIV co-morbidities | Secondary | 138 |
| *How do A-bies exercise?* | Daily life | Primary | 145 |
| *Can I have sex with A-bies without a condom?* | Transmission prevention | Secondary | 152 |
| *Healthy life: tips about protecting your liver* | Daily life | Primary | 159 |
| *Simple ways to enhance your immune function* | Daily life | Primary | 173 |
| *Love yourself: seven ways to make you feel good.* | Psychological adaptation | Secondary | 180 |

A: HIV/AIDS; A-bies: Alias for people living with HIV/AIDS to protect privacy.

# Table S3. Delivery arrangement of instruction messages on service retrieval within the platform

| **Function** | **Message content** | **Days of delivery** |
| --- | --- | --- |
| Introduction | *Welcome my friend! In the next six months, we will accompany you through the initial six months of your antiviral treatment. During this process, you will receive the following messages/articles: (i) HIV-related knowledge; (ii) reminders for follow-up; (iii) project questionnaires; in addition, you will be able to communicate online with your case manager on this platform and get information about articles or services that interest you through the platform menu. We hope we will be able to help you adapt to the treatment. Thank you for your participation and wish you good health!* | 0 (enrolment) |
|  |  |  |
| Communication reminder | *How do you feel as the week goes by? If you have questions you can reply to this message to contact us.* | 7 |
|  | *It's been two weeks since the start of the new trip, how are you adjusting to it? If you have questions you can reply to this message to contact us.* | 14 |
|  |  |  |
| Visit reminder | *Please remember to pick up your medication in a week.”* | 24,54,82,173 |
|  |  |  |
| Instruction to retrieve supportive info | *Hello, in addition to the fixed push content, you can 1) communicate with follow-up nurses; 2) get articles on topics of interest; 3) get other service content through the menu bar at the bottom of the public number at any time.* | 31,61,91,121,151 |
|  |  |  |
| Questionnaire reminder | *Good evening, you've been taking your medication for xx month now, how have you been feeling during this time? Please fill out this questionnaire when you have time so we can get to know you better.* | 30,90,180,360 |

# Table S4. Definition and reporting for process evaluation indicators

| **Indicators** | **Data source** | **Process evaluation indicators** | | | |
| --- | --- | --- | --- | --- | --- |
|  |  | **Dose delivered** | **Dose received** | **Fidelity** | **Satisfaction** |
| Online communication | Trusted Doctor |  | $\surd$ | $\surd$ | $\surd$ |
| Educational article delivery | Trusted Doctor | $\surd$ * | $\surd$ |  |  |
| Supportive service information retrieval | WeChat | $\surd$ * | $\surd$ |  |  |
| Hospital visit reminders | WeChat | $\surd$ * |  |  |  |

^*^: Educational articles delivery, instructive message for supportive service information retrieval, and hospital visit reminders were automatically sent by the app, and their dose delivered were identical for each participant and were described in Methods.

# Figure S2. Online patient-provider communication framework

**Background variables**

• Socio-demographics

• Psycho-social status

**Conversation feature**

• Conversation content

**Online features**

• Completeness

• Timeliness

• Style

**Outcome**

• Adherence

• Satisfaction

• Physical health

• Mental health

# Table S5. Coding rules for online communication data

| **Coding rule** | **Categories** | **Definition** |
| --- | --- | --- |
| Content | Instrumental | Medication taking and ART-relevant problem solving |
|  | - Side effect | - related to side effect of ART |
|  | - Medication taking behavior | - Related to the aftermath of inadherence and approaches to remedy |
|  | - HIV and ART related knowledge | - Difference between regimens |
|  | - Physical examination | - Physical examination to take, how to interpret test results |
|  | - Hospital visits process | - Documents/process for hospital follow-ups |
|  | - Transmission prevention | - How to prevent transmission to family/friends/partner |
|  | - Daily life | - If certain daily behaviors (e.g., Food/drink intake, exercise, sleep) will affect treatment outcome |
|  | - HIV co-morbidities | - co-morbidities including sexually transmitted illness, Hepatitis B Virus infection, etc |
|  | - Other | - Not belonging to the above categories |
|  | Affective | socio-emotional expression or support |
|  | - Patients express negative emotions | - patients explicitly expressed negative emotions |
|  | - Case managers provide emotional support | - case managers provide emotional support/comfort |
| Timeliness | Getting replied within 2 hours | The time between the initiating message and the first reply message was less than 2 hours |
|  | Getting replied within 24 hours | The time between the initiating message and the first reply message was less than 24 hours |
| Completeness | Not getting any reply | There is no reply to the messages initiating dialogue |
|  | Replied | There is reply to the messages initiating dialogue |
|  | - Solved clearly and directly | - answers/solutions to questions were provided directly |
|  | - Referred to other professionals | - the patients were suggested to ask other professionals (e.g., physicians) |
|  | - Unsolved | - the case managers answered but didn’t provide direct solution or suggest professionals to turn to. |
| Style | Using emoticons | Emoticons involved in conversation |
|  | - Patients using emoticons | -Emoticons involved in patients-sent messages |
|  | - Case managers using emoticons | -Emoticons involved in case managers-sent messages |
|  | Mannered languages | Mannered languages involved in dialogues |
|  | - Patients using polite languages | -Mannered languages involved in patients-sent messages |
|  | - Case managers using polite languages | -Mannered languages involved in case managers-sent messages |

1. Minimal unit of online conversation coding: dialogue.
2. Definition of dialogue: Intended interaction focusing on one topic/event initiated by either side with text-based messages regardless of getting replied.
3. Eligibility criteria for online communication data cleaning: (i). message contains text (Excluding messages consisted of web-link or emoticons only). (ii). messages sent by patients or case managers (Excluding automatically messages sent by app).

Table S6. Baseline and month-one Information Motivation and Behavioral skills model constructs in intervention and control group.

| **Outcomes** | **All** | **Control group** | **Intervention group** | **P value** |
| --- | --- | --- | --- | --- |
| Baseline IMB scores |  |  |  |  |
| Information score | 39.3 (4.46) | 39.4 (4.47) | 39.2 (4.46) | 0.708 |
| Motivation score | 32.9 (7.93) | 33.2 (8.40) | 32.7 (7.45) | 0.616 |
|  |  |  |  |  |
| Month-1 outcomes |  |  |  |  |
| Information score | 37.5 (4.98) | 37.7 (4.67) | 37.3 (5.26) | 0.483 |
| Motivation score | 32.4 (8.31) | 31.6 (8.48) | 33.2 (8.10) | 0.110 |
| Behavioral skills | 50.3 (9.51) | 50.1 (9.60) | 50.5 (9.46) | 0.759 |


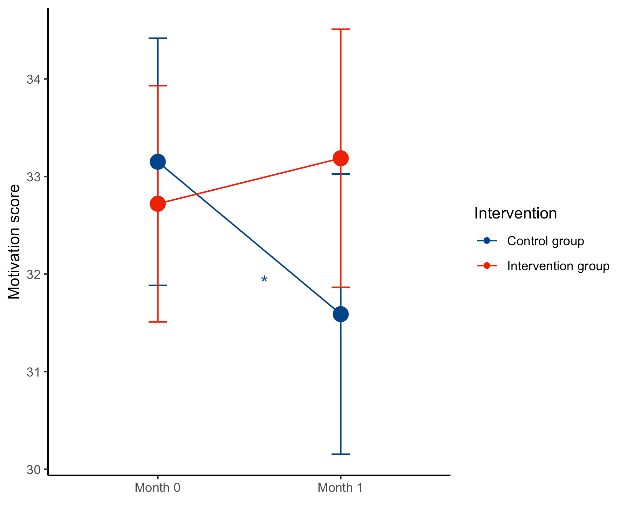

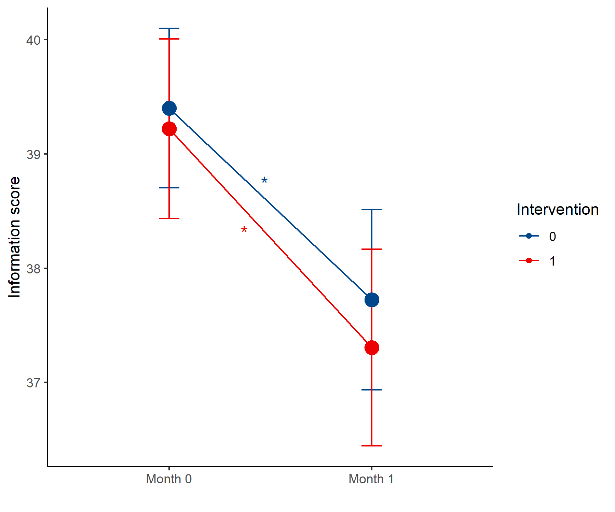
Figure S3. Changes of information and motivation scores from baseline to month one in two groups. * star sign indicated a significant decrease from baseline to month-one follow-up.

B

A

Figure S4. Results of structural equation modeling to test the IMB framework in the full sample. * star sign indicated significant associations.

Information score

Motivation score

Behavioural skills

Adherence

0.29 (0.19, 0.38)*

0.49 (0.39, 0.59)*

0.99 (0.60, 1.38) *

0.24 (-0.08, 0.55)

-0.11 (-0.46, 0.23)

Table S7. Association between intervention and contamination and outcomes. Education level was adjusted due to the unbalanced distribution in the intervention and control group. Baseline information score and motivation score were adjusted respectively.

| Outcomes | Subscribing to another article source | Communicated with research assistants |
| --- | --- | --- |
| **Health-related outcomes** | Odds ratio (95% CI) | |
| Adherence |  |  |
| Control group without contamination | Ref | Ref |
| Control group with contamination | 0.84 (0.41, 1.75) | 1.18 (0.56, 2.48) |
| Intervention group | 1.16 (0.61, 2.2) | 1.38 (0.80, 2.37) |
| **Intermediate outcomes** |  |  |
| Information score | Beta coefficients (95% CI) | |
| Control group without contamination | Ref | Ref |
| Control group with contamination | 0.36 (-1.17, 1.90) | **-1.8 (-3.37, -0.22)** |
| Intervention group | 0.15 (-1.23, 1.53) | -0.72 (-1.96, 0.53) |
| Motivation score |  |  |
| Control group without contamination | Ref | Ref |
| Control group with contamination | 0.33 (-1.96, 2.61) | -0.67 (-3.01, 1.68) |
| Intervention group | **2.75 (0.73, 4.78)** | **2.2 (0.58, 3.83)** |
| Behavioral skills |  |  |
| Control group without contamination | Ref | Ref |
| Control group with contamination | 0.23 (-3.16, 3.63) | -1.91 (-5.24, 1.42) |
| Intervention group | 1.11 (-1.75, 3.97) | 0.46 (-1.87, 2.80) |

Table S8. Association between baseline variable and month one adherence in the control group. Univariable models were constructed to obtain odds ratio (OR) and 95% confidence interval. NA: not applicable.

| **Variable** | **Events** | **Respondents** | **%** | **OR (95% CI)** | **P value** |
| --- | --- | --- | --- | --- | --- |
| Age |  |  |  |  |  |
| 18~24 | 12 | 36 | 33.3 | Ref |  |
| 24~30 | 22 | 48 | 45.8 | 1.95 (0.79, 4.80) | 0.149 |
| >=30 | 21 | 47 | 44.7 | 1.89 (0.75, 4.77) | 0.182 |
| Education |  |  |  |  |  |
| Middle school or lower | 4 | 18 | 22.2 | Ref |  |
| High school | 6 | 21 | 28.6 | 1.61 (0.38, 6.89) | 0.521 |
| College degree | 26 | 51 | 51 | **3.87 (1.20, 12.47)** | **0.025** |
| Bachelor’s degree or above | 21 | 44 | 47.7 | **3.64 (1.14, 11.68)** | **0.031** |
| Employment |  |  |  |  |  |
| Students | 9 | 22 | 40.9 | Ref |  |
| Public sector | 2 | 4 | 50 | 1.32 (0.16, 11.08) | 0.800 |
| Private sector | 31 | 64 | 48.4 | 1.68 (0.63, 4.47) | 0.304 |
| Other | 15 | 44 | 34.1 | 0.78 (0.28, 2.22) | 0.645 |
| Monthly income |  |  |  |  |  |
| <1,000 | 6 | 24 | 25 | Ref |  |
| 1,000-5,000 | 13 | 30 | 43.3 | 2.52 (0.79, 8.06) | 0.122 |
| 5,000-10,000 | 21 | 49 | 42.9 | 2.68 (0.92, 7.83) | 0.072 |
| >10,000 | 17 | 31 | 54.8 | **4.51 (1.43, 14.23)** | **0.011** |
| Marital status |  |  |  |  |  |
| Single | 34 | 74 | 45.9 | Ref |  |
| Not single | 23 | 60 | 38.3 | 0.67 (0.35, 1.30) | 0.240 |
| Sexual orientation |  |  |  |  |  |
| Homosexual/Bisexual | 52 | 118 | 44.1 | Ref |  |
| Heterosexual or unknown | 5 | 16 | 31.2 | 0.55 (0.18, 1.68) | 0.295 |
| Baseline CD4 count |  |  |  |  |  |
| >=350 | 27 | 69 | 39.1 | Ref |  |
| <350 | 30 | 65 | 46.2 | 1.38 (0.70, 2.70) | 0.352 |
| Regimen |  |  |  |  |  |
| Free first-line regimen | 48 | 114 | 42.1 |  |  |
| Other regimens | 9 | 20 | 45 | 1.14 (0.46, 2.85) | 0.775 |

Table S9. Association between baseline variable and month one month-one Information-Motivation-Behavioral skills (IMB) model constructs in the control group. For information scores, whether engaged in online communication was adjusted.

| **Variable** | **Information** |  | **Motivation** |  | **Behavioral skills** |
| --- | --- | --- | --- | --- | --- |
| Age |  |  |  |  |  |
| 18~24 | Ref |  | Ref |  | Ref |
| 24~30 | 1.92 (-0.18, 4.02) |  | 1.68 (-2.09, 5.45) |  | 3.48 (-0.73, 7.68) |
| >=30 | 0.60 (-1.51, 2.7) |  | -0.31 (-3.94, 3.32) |  | 2.54 (-1.9, 6.98) |
| Education |  |  |  |  |  |
| Middle school or lower | Ref |  | Ref |  | Ref |
| High school | 0.34 (-2.74, 3.42) |  | -3.78 (-9.01, 1.44) |  | -3.32 (-9.79, 3.15) |
| College degree | -0.09 (-3.15, 2.97) |  | -3.49 (-7.51, 0.54) |  | 0.01 (-5.12, 5.15) |
| Bachelor’s degree or above | 1.82 (-0.91, 4.54) |  | -2.43 (-6.97, 2.12) |  | 1.00 (-4.04, 6.04) |
| Employment |  |  |  |  |  |
| Students | Ref |  | Ref |  | Ref |
| Public sector | -1.01 (-4.93, 2.91) |  | 3.1 (-5.20, 11.41) |  | 2.73 (-6.06, 11.53) |
| Private sector | 0.08 (-2.28, 2.44) |  | 0.38 (-3.78, 4.54) |  | 1.29 (-3.12, 5.71) |
| Other | -1.31 (-3.75, 1.14) |  | -0.57 (-4.99, 3.85) |  | -3.08 (-7.99, 1.83) |
| Monthly income |  |  |  |  |  |
| <1,000 | Ref |  | Ref |  | Ref |
| 1,000-5,000 | -0.47 (-3.12, 2.18) |  | 2.05 (-2.13, 6.22) |  | 3.27 (-1.74, 8.28) |
| 5,000-10,000 | -0.61 (-3.04, 1.81) |  | 0.32 (-3.95, 4.59) |  | 3.26 (-1.17, 7.69) |
| >10,000 | -0.03 (-2.62, 2.56) |  | -1.5 (-5.92, 2.92) |  | 2.15 (-2.90, 7.19) |
| Marital status |  |  |  |  |  |
| Single | Ref |  | Ref |  | Ref |
| Not single | -0.43 (-2.03, 1.17) |  | 0.92 (-2.17, 4.01) |  | 0.12 (-3.21, 3.46) |
| Sexual orientation |  |  |  |  |  |
| Homosexual/Bisexual | Ref |  | Ref |  | Ref |
| Heterosexual or unknown | 0.93 (-1.53, 3.40) |  | -0.4 (-4.48, 3.68) |  | -1.98 (-7.01, 3.05) |
| Baseline CD4 count |  |  |  |  |  |
| >=350 | Ref |  | Ref |  | Ref |
| <350 | 0.37 (-1.16, 1.90) |  | 1.36 (-1.51, 4.22) |  | 1.89 (-1.22, 5.01) |
| Regimen |  |  |  |  |  |
| Free first-line regimen | Ref |  | Ref |  | Ref |
| Other regimens | -1.71 (-4.04, 0.62) |  | -1.64 (-5.61, 2.34) |  | -0.33 (-4.93, 4.27) |
